# Oral Pathology in COVID-19 and SARS-CoV-2 Infection—Molecular Aspects

**DOI:** 10.3390/ijms23031431

**Published:** 2022-01-27

**Authors:** Agnieszka Drozdzik, Marek Drozdzik

**Affiliations:** 1 Department of Integrated Dentistry, Pomeranian Medical University in Szczecin, Powstancow Wlkp 72, 70-111 Szczecin, Poland; agnieszka.drozdzik@pum.edu.pl; 2 Department of Pharmacology, Pomeranian Medical University in Szczecin, Powstancow Wlkp 72, 70-111 Szczecin, Poland

**Keywords:** COVID-19, SARS-CoV-2, oral cavity

## Abstract

This review article was designed to evaluate the existing evidence related to the molecular processes of SARS-CoV-2 infection in the oral cavity. The World Health Organization stated that severe acute respiratory syndrome coronavirus 2 (SARS-CoV-2) infection and transmission is produced by respiratory droplets and aerosols from the oral cavity of infected patients. The oral cavity structures, keratinized and non-keratinized mucosa, and salivary glands’ epithelia express SARS-CoV-2 entry and transmission factors, especially angiotensin converting enzyme Type 2 (ACE2) and transmembrane serine protease 2 (TMPRSS2). Replication of the virus in cells leads to local and systemic infection spread, and cellular damage is associated with clinical signs and symptoms of the disease in the oral cavity. Saliva, both the cellular and acellular fractions, holds the virus particles and contributes to COVID-19 transmission. The review also presents information about the factors modifying SARS-CoV-2 infection potential and possible local pharmacotherapeutic interventions, which may confine SARS-CoV-2 virus entry and transmission in the oral cavity. The PubMed and Scopus databases were used to search for suitable keywords such as: SARS-CoV-2, COVID-19, oral virus infection, saliva, crevicular fluid, salivary gland, tongue, oral mucosa, periodontium, gingiva, dental pulp, ACE2, TMPRSS2, Furin, diagnosis, topical treatment, vaccine and related words in relevant publications up to 28 December 2021. Data extraction and quality evaluation of the articles were performed by two reviewers, and 63 articles were included in the final review.

## 1. Introduction

This review article was designed to evaluate the existing evidence related to the molecular processes of SARS-CoV-2 infection in the oral cavity (the reference search protocol in provided in Supplementary Materials). Clinical signs of severe acute respiratory syndrome coronavirus 2 (SARS-CoV-2) infection such as dry mouth, loss of taste and mucosal lesions (ulcerations, enanthema and macules) can be observed in about half of coronavirus disease 2019 (COVID-19) subjects. Recent observations suggest that the oral cavity may not only be a site of the disease’s clinical manifestation but also can play a substantial role in entry and transmission of the SARS-CoV-2 virus. There is growing evidence that the virus can directly infect and replicate in oral tissues, i.e., the mucosa and salivary glands. A clinical picture of the pathological states of the oral cavity in COVID-19 has been recently presented in several reviews [1,2].

The SARS-CoV-2 can enter cells via two pathways, i.e., through host membrane-bound peptidases or endocytosis. The principal entry mechanism is initiated by binding of the spike protein (S) of the viral envelope, mainly to the SARS-CoV-2 entry factor, i.e., angiotensin-converting enzyme 2 (ACE2), which is a metallopeptidase harbored on the cell membrane. The spike is cleaved into S1 and S2 by a host cell-derived protease, which is assumed to be furin. The S1 subunit dissociates from the rest of the spike protein and S2 is cleaved by host cell-derived transmembrane serine protease 2 (TMPRSS2), which exposes the fusion peptide for host cell membrane fusion and cell invasion. In vitro studies also suggest the potential role of neuropilin-1 (NRP-1), a member of a family of signaling proteins, which have been shown to serve as an entry factor and potentiate SARS-CoV-2’s infectivity [3]. Other tissue-specific proteases (TMPRSS4 and TMPRSS11D) and endosomal proteases (CTSB, CTSL, BSG) are also known to mediate entry of the virus for intracellular replication [4]. The endosomal pathway is initiated by the interaction of the SARS-CoV-2 spike protein with the host cell receptor ACE2 located on the cell membrane, with subsequent endocytosis of the virus. The spike protein is processed by cathepsin L to cleave into S1 and S2 in the endosome, which allows fusion of the viral capsid with the endosomal membrane. The endosomal processing results in release of the virus genome. However, ACE2 and TMPRSS2 are critical factors in the infection process of SARS-CoV-2 [5].

## 2. Oral Cavity as the SARS-CoV-2 Entry Gateway

### 2.1. Oral Mucosa

Expression of the major SARS-CoV-2 entry factors, i.e., ACE2 and TMPRSS (along with others), was identified in the oral mucosa, tongue and salivary glands [6]. The oral cavity mucosa is lined by stratified squamous epithelia, divided into keratinized (gingiva and hard palate) and non-keratinized (buccal, labial, ventral tongue and oropharyngeal) mucosa. In the buccal mucosa (non-keratinized), the expression of ACE2 was observed in the cytoplasm and on the cell membrane, and TMPRSS2 was found on the cell membrane by using immunolocalization to visualize the proteins. In the mucosal structures, a very weak expression of ACE2 was noticed in the lamina propria of the non-keratinized stratified squamous epithelia, whereas TMPRSS2 was not observed in the stratum basale or in the lamina propria. The non-keratinized stratified squamous epithelia of the labial mucosa harbor both ACE2 (in the cytoplasm and on the cell membrane) and TMPRSS2 (on the cell membrane). Similar to the buccal mucosa, very weak expression of ACE2 in the lamina propria was observed, while the expression of TMPRSS2 in the stratum basale or in the lamina propria was not noted [7]. The keratinized stratified squamous epithelia demonstrate ACE2 immunolocalization in the cytoplasm and on the cell membrane, mainly in the stratum granulosum of the epithelia, while TMPRSS2 is strongly expressed on the cell membrane, mainly in the stratum granulosum and stratum spinosum, but not in the stratum basale [7]. Another immunohistochemical analysis corroborated the aforementioned observations, and showed ACE2 immunolocalization in the stratified squamous epithelium of the gingiva and that of TMPRSS2 in the stratified squamous epithelium in the keratinized surface layer. This study also reported the localization of furin, mainly in the lower layer of stratified squamous epithelia [8]. Okui et al. confirmed ACE2 immunolocalization in gingival cells collected from the gingival sulcus (and quantified to the same level as the tongue, which was formerly considered as one on the main SARS-CoV-2 entry routes, along with the salivary glands). The expression level of ACE2 in the keratinized mucosa of the palate was documented to be very mild [9].

More advanced immunohistochemistry methods support the expression pf SARS-CoV-2’s entry and transmission molecules in the oral cavity. A single-cell RNA sequencing analysis demonstrated that multiple oral epithelial cell subtypes express the virus’s entry factors. Mucosal keratinocytes expressed ACE2 and TMPRSS2 as well as the endosomal proteases CTSB and CTSL [10]. The single-cell sequencing data of Xu et al. further proved the expression of the ACE2 receptor in the buccal mucosa and gingiva [11]. The normalized ACE2 and TMPRSS2 co-expression levels in the oral mucosa were similar to known sites of infection by SARS-CoV-2, i.e., nasal and intestinal epithelial cells [12,13].

In situ hybridization assays in healthy adult tissue samples from the buccal mucosa, soft palate and palatine also documented increased suprabasal expression of ACE2 and TMPRSS2 compared with the basal compartment (non-keratinized) [10].

These results point to multiple sites in the oral mucosa as potentially susceptible to infection by SARS-CoV-2, which makes it prone to the infection with a possibility of viral transmission to the respiratory and gastrointestinal tract (see below). The expression of ACE2 and TMPRSS2 in the sulcular epithelium and periodontal pocket epithelium, along with the microenvironment of periodontal pockets, crevices and the gingival sulcus, may also provide favorable conditions for virus replication and maintenance.

Colonization of the oral mucosa by SARS-CoV-2 was demonstrated by Huang et al. [10]. The virus spike was independently localized both inside the shed epithelial cells and on their membrane surface. This study also revealed infection and viral replication in all layers of mucosa (primarily enriched in the differentiated epithelial cells). Furthermore, mucosal scrapings demonstrated features of infection and replication capacity after shedding. Thus, these cells can play the role of virus carriers, and promote viral stability and transmissibility.

### 2.2. Tongue

Gustatory dysfunction has been reported in about 40% of COVID-19 patients, being most commonly diagnosed between 2 and 14 days after exposure to SARS-CoV-2 [1,2]. Expression of SARS-CoV-2 entry factors in taste papillae cells allows virus invasion with subsequent cell damage and a clinical manifestation of dysgeusia (see the details below).

In the lingual mucosa, the expression of both ACE2 and TMPRSS2 using immunohistochemistry was observed in the keratinized stratified squamous epithelia. The expression of ACE2 was shown in the cytoplasm and on the cell membrane, and very weakly in the lamina propria, whereas the immunolocalization of TMPRSS2 was determined on the cell membrane but not in the stratum basale or in the lamina propria [7]. The immunohistochemistry staining reported by Sakaguchi et al. [8] confirmed the aforementioned findings of Sawa et al. [7]. This study demonstrated ACE2–TMPRSS2 co-expression in cell membranes of the keratinized squamous epithelial cells of the dorsal tongue. Immunohistochemical analysis also demonstrated ACE2 localization in the nucleus and the weak expression and localization of TMPRSS2 in the cytoplasm of the papillae taste cells [8]. RNA analysis of human fungiform papillae taste cells confirmed expression of ACE2 and TMPRSS2 [8].

Hybridization in situ analysis was in line with the latter study, as the strong expression of ACE2 and the low expression of TMPRSS2 in suprabasal (superficial) and basal cell populations of the ventral and dorsal tongue were determined. The single-cell sequencing data of Xu et al. further proved the expression of the ACE2 receptor in the tongue, which is highly enriched in epithelial cells [11]. In the dorsal tongue, the squamous epithelium is thick due to stratification of the cells, and it is unclear whether the cells in the superficial layer can mediate the infection of subepithelial cells.

Similar to mucosa, the expression of SARS-CoV-2 entry factors can open the virus infection gateway. In fact, infection with the virus was determined in the dorsal tongue lining from autopsy patients [10], as well as in SARS-CoV-2-infected subjects. This finding corroborates observations from cytological smears from the dorsum of the tongue, which revealed that 71% of the COVID-19 patients presented epithelial cells positive for the presence of the SARS-CoV-2 spike protein [14].

### 2.3. Salivary Glands

The salivary gland epithelia seem to be the main entry gateway for SARS-CoV-2 infection. Higher levels of the virus entry factors were found than in other epithelial cells in the oral cavity. An immunohistochemical assay revealed the expression of both ACE2 and TMPRSS2 in the labial gland within the mucous and serous acini. The stronger expression of TMPRSS2 in the serous acini than in the mucous acini was observed. In the striated ducts, ACE2 was also found, while TMPRSS2 was not noted. These findings suggest that the virus primarily attaches to the oral mucosa and the orifice of the salivary glands’ ducts, and also in the minor salivary glands distributed all over the oral mucosa [7]. The cellular localization of the SARS-CoV-2 entry factors was reported by Zhu et al., who provided information about the major salivary glands from patients with benign disorders. Immunohistochemistry revealed ACE2 and TMPRSS2 protein localization in the cytoplasm and cytomembrane of serous acinar cells and duct epithelial cells of the parotid and submandibular glands, and in the cytoplasm and cytomembrane of serous acinar cells in the mixed acini of sublingual glands. Protein quantification using Western blot analysis revealed decreasing abundance levels of ACE2 and TMPRSS2 in the order of parotid, submandibular and sublingual glands (especially observed for TMPRSS2) [15].

Single-cell RNA sequencing, supported by in situ hybridization, also demonstrated the co-expression of ACE2 and protease TMPRSS2 (as well as other proteases, CTSB and CTSL) in the epithelial cells of the salivary glands (parotid, submandibular and labial minor). This study also demonstrated different tissue-specific expression patterns of proteases, with TMPRSS2 enriched in the salivary glands’ epithelia and TMPRSS11D enriched in mucosal keratinocytes. Different patterns of proteases may suggest a tissue-specific infection gateway [10]. The endosomal proteases CTSB and CTSL exhibited broad expression patterns across epithelia. The expression levels of the entry factors in the salivary glands were higher (especially in the minor salivary glands) than in the oral cavity mucosa; more importantly, co-expression of the principal entry factors ACE2 and TMPRSS2, especially in the acini and duct epithelial cells, was observed. Moreover, the viral entry factor expression levels in the minor salivary glands were found to be similar to those of the known virus gateways in the gastrointestinal and respiratory tracts [16].

The SARS-CoV-2 invasion potential was confirmed by an experimental study reported by Zhu et al., who revealed that the SARS-CoV-2 spike proteins were able to bind in salivary gland homogenates to human parotid, submandibular and sublingual gland cells [15]. Similar to the oral mucosa, both the minor and major salivary glands were found to exhibit replicating SARS-CoV-2. Huang et al., using minor salivary glands from an individual acutely infected with COVID-19 and from autopsies, revealed replicated SARS-CoV-2 in infected acini and ducts. Parotid salivary glands also revealed infection but to a lesser degree [10]. SARS-CoV-2 infection of the submandibular glands was reported by Schurink et al. [17] and Matuck et al. [18]. The study of postmortem biopsies of fatal COVID-19 cases revealed the positive detection of SARS-CoV-2 RNA in 60% of submandibular and parotid gland specimens as spherical 70–100 nm viral particles (consistent in size and shape with the Coronaviridae family) observed through electron microscopic analysis [18].

### 2.4. Dental Pulp

A global transcriptomic analysis reported that the SARS-CoV-2 entry factors, i.e., ACE2 and TMPRSS2, are expressed in healthy and inflamed human dental pulp without significant differences between healthy control and diseased biopsies [19]. These findings were confirmed by Altaie et al. in an RNA expression study, which revealed the expression of ACE2, TMPRSS2 and NRP1 in healthy pulp tissues [20]. It can be suggested that healthy and inflamed pulp tissues have a similar tendency to be infected by SARS-CoV-2.

### 2.5. Factors Affecting SARS-CoV-2 Entry Factors’ Expression and the Virus Infection Potential

Clinical observations suggest that the expression of the virus entry factors can be both age- and sex-dependent. Peng et al. [21] revealed that the mRNA expression levels of ACE2 and TMPRSS2 in the oral mucosa were significantly increased in relatively elderly women and men (>50–60 years of age) than in relatively younger individuals (both females and males). The expression levels of ACE2 in oral tissues was comparable in males and females of similar age, whereas the mRNA expression of TMPRSS2 was lower in female oral tissues than that in age-matched males. ACE2 and TMPRSS2 protein level analysis using Western blotting and immunohistochemistry confirmed the RNA findings, i.e., higher levels in the mucosa of the elderly than in those of younger people. However, no significant proteomic differences between females and males at a similar age were observed. Bioinformatic analysis of ACE2 and TMPRSS2 in different cohorts (TCGA, GSE42743, GSE9844, GSE30784 databases) also supported observations of increased expression of both ACE2 and TMPRSS2 with advancing age. A tendency for higher levels of the virus entry factors in male oral epithelia compared with those from females also appeared. Higher expression levels of ACE2 and TMPRSS2 in male and older cohorts coincided with a higher positive rate of SARS-CoV-2 in the saliva [21]. Based on these findings, the elderly population might be more prone to SARS-CoV-2 infection through oral transmission. This statement is supported by clinical observations, demonstrating higher susceptibility to infection and clinical symptom manifestation in elderly populations [22].

Local inflammatory process in the oral cavity may modulate SARS-CoV-2 entry. In vitro studies demonstrated that factors involved in the pathogenesis of periodontal diseases, e.g., *Porphyromonas gingivalis,* and cytokines may impact SARS-CoV-2’s entry and processing molecule expression. Exposure of human gingival fibroblasts to *Porphyromonas gingivalis*-derived lipopolysaccharide (PgLPS) or IL1β, TNFα and PGE2 resulted in significant elevation of ACE2. Likewise, expression of TMPRSS2 was increased by some inflammatory mediators, i.e., PgLPS, IL1β, or PGE2. The expression of furin decreased after TNFα treatment [23]. These findings suggest that local inflammation in the periodontal gingiva may increase the risk of virus infection. It can be also considered that the lytic activity of periodontal bacteria can produce synergistic activity with membrane proteases, which might favor early and prolonged SARS-CoV-2 colonization of the oral cavity [24,25]. These ideas may be supported by findings on detection of the virus in saliva samples before the development of clinical symptoms [26] and persisting for a period of time even after symptom relief [27]. Likewise, Marouf et al., based on a case–control study, reported an association between periodontitis and the severity of COVID-19 infection. The report revealed that periodontitis was associated with a higher risk of intensive care unit admission, demand for assisted ventilation and death in COVID-19 patients [28].

The response of periapical tissues to inflammatory factors, including IL-6, seems to be opposite to the periodontal tissues cited above. Altaie et al. [20] indicated that the expression of ACE2 and TMPRSS2 (also NRP-1) was significantly reduced in oral periapical lesions (periapical abscesses, preapical granulomas, radicular cysts) compared with healthy pulp tissues. A reverse correlation between IL6 and ACE2, NRP1 and TMPRSS2 gene expression in periapical abscesses and granuloma was noted. However, Galicia et al. [19] reported similar expression levels of both ACE2 and TMPRSS2 in tissues from normal and inflamed dental pulp. These findings are similar to the expression patterns in other tissues [29,30], and suggest that ACE2 and TMPRSS2 remain unchanged under inflammatory conditions.

Another factor present in saliva which can modulate SARS-CoV-2 infection potential is the SARS-CoV-2 spike 1 cross-reactive IgA. The IgA was shown to partly suppress the binding of the SARS-CoV-2 spike protein to ACE2 receptors. The immunoglobulin was detected in nearly half of individuals who had never been infected with the virus, with decreasing levels in those aged over 50 years (IgA levels decrease with age). These antibodies belong to cross-reacting antibodies of other homologous coronaviruses. Their presence in the saliva suggest that the IgA may help prevent SARS-CoV-2 infection, and decreased levels in elderly subjects may contribute to the higher virus infective potential and COVID-19 incidence in this population [31]. Those results corroborate the clinical findings of a less frequently severe (and often asymptomatic) COVID-19 course in children and adolescents [32,33]. IgA antibodies against SARS-CoV-2, which constitute the early specific humoral response, were also detected in COVID-19 patients and were shown to persist in saliva for 49–73 days after the occurrence of symptoms [34]. These antibodies can be considered as a specific defense mechanism and potentially could be explored as a potential diagnostic parameter.

Environmental factors can also be involved in promoting SARS-CoV-2 infection in the oral cavity. A recent study has documented that smoking can increase the susceptibility to COVID-19 disease. It was found that cigarette smoke condensates upregulated ACE2 and TMPRSS2 expression in human gingival epithelial cells. Moreover, this study provided evidence that exposure to cigarette smoke condensates potentiated the internalization of a SARS-CoV-2 pseudovirus in the cells in an AhR-dependent manner (AhR is a nuclear receptor that is a known mediator of cigarette smoke responses) [35]. Thus, the experimental findings suggest that smoking cessation could reduce the susceptibility to coronavirus infection of the oral cavity structures.

**Figure 1:**
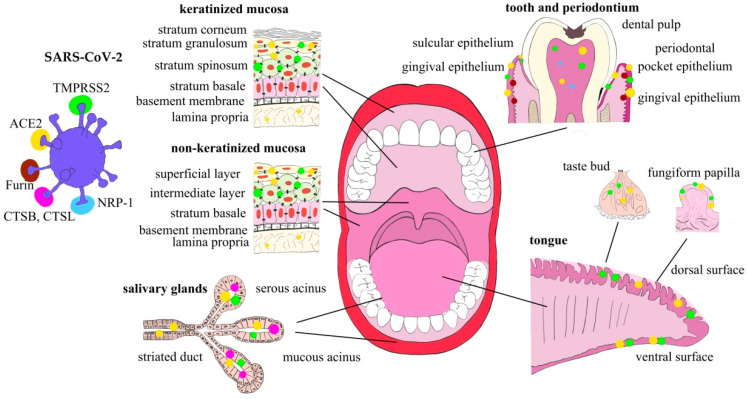
Expression of SARS-CoV-2 entry and transmission factors in the oral cavity structures.

Finally, there is a very preliminary observation that the SARS-CoV-2 virus itself can modulate the expression of ACE2 in the oral mucosa, as buccal mucosa smear samples from patients with COVID-19 demonstrated downregulation of ACE2 mRNA [36]. Therefore, it can be hypothesized that the cellular response to SARS-CoV-2 infection has defensive characteristics protecting a cell from the viral particle overload (Expression of SARS-CoV-2 entry and transmission factors in the oral cavity are summarized in Figure 1). 

## 3. Saliva as a Potential Transmission Factor

Respiratory droplets originating from the nose, oral cavity and airways constitute one of the principal transmission routes of SARS-CoV-2 [37], and saliva is one of the most important sources of droplets. The SARS-CoV-2 viral RNA load in the oral fluid globally ranges from 9.9 × 10^2^ to 7.1 × 10^10^ copies/mL, peaking in the first week of symptom onset and declining over time with recovery.

Oral mucosa and salivary gland cells can be shed and further detected in the saliva. Huang et al. reported that ~5–10% of all salivary cells (pan-cytokeratin positive, pCK+) were infected by SARS-CoV-2 in saliva samples collected from mildly symptomatic individuals [10]. Viral replication inside those shed salivary epithelial cells has been observed, which could promote the spread of infection and increase the transmission potential of saliva. Similarly, suprabasal mucosal cells showed expression of the virus entry factors and evidence of SARS-CoV-2 infection. These cells are shed from the most terminally differentiated tissue layers about every 3 h and can constitute the virus transmission mechanism, but the shedding might also serve as a potential local protective mechanism against oral tissue infection [38]. These cells were revealed to transmit the virus to Vero cells ex vivo from saliva with a high viral load [10]. Moreover, a rare population of pCK+ (i.e., ciliated) cells (typically found in the respiratory tract) that were SARS-CoV-2-positive were detected in saliva. This finding indicated the contribution of the respiratory tract cell population to saliva’s viral load, which may play a role in SARS-CoV2 infection propagation in the oral cavity and sustain COVID-19 in other body sites [39]. Moreover, the infected exfoliated epithelial cells in saliva may be involved in the infection spreading to the lower respiratory tract and/or gastrointestinal tract.

Not only infected cells but also cell-free saliva can constitute an infectious medium, as it has been proven that SARS-CoV-2 RNA-positive saliva from asymptomatic individuals was able to induce a cytopathic effect typical of coronavirus infection, along with virus replication [10,40]. The viral particles from supernatants of cultures a the cytopathic effect were able to demonstrate infectious potential in new cell monolayers. These findings reveal the infectious SARS-CoV-2 potential of saliva, i.e., expelled oral droplets containing infectious virus and infected cells, even from asymptomatic/pre-symptomatic subjects, are a source of airborne transmission. The saliva can be cleared of SARS-CoV-2 over very long periods. In asymptomatic subjects, it takes from 0.5 weeks to 3.5 weeks (from the first test to the negative test), and in individuals with symptoms, it takes about 1 week longer [10]. Some observations have also suggested that the virus can be cleared from the nasopharynx but still can be present in the saliva, which may suggest sustained shedding of the virus from SARS-CoV-2 infected oral sites [10].

SARS-CoV-2 virus replication has been observed in the periodontium, from where the virus can reach not only the oral cavity and saliva but can also spread to the bloodstream of the local periodontal capillary network and further progress to distant organs. Thus, the oral cavity can not only contribute to external infectious potential but may also promote development and recurrence of the systemic disease [25,41]. Recently, a postmortem study demonstrated SARS-CoV-2 RNA in the periodontal tissues of COVID-19-positive patients several days (up to 24 days) after the onset of first symptoms, with possible virus presence in the crevicular fluid [42]. It can be suggested that the periodontal pocket could act as a favorable niche or reservoir for both active and latent SARS-CoV-2 forms. Collectively, these findings demonstrate that the oral cavity is an important site for SARS-CoV-2 infection, and implicate saliva, both the acellular and cellular fractions, as a potential route of SARS-CoV-2 transmission [10].

Public health measures, such as universal mask use and social distancing, are dedicated to reducing droplet and aerosol transmission. The effectiveness of standard mask wearing to reduce droplet demonstrated a more than 10-fold decrease in the detection of expelled salivary droplets, including in some asymptomatic individuals with a positive nasopharyngeal or saliva viral load [10].

Dental practitioners can be, in additionally to the abovementioned factors of saliva being a risk for the general population, exposed during routine oral cavity-related procedures to other infectious material. The SARS-CoV-2 entry and transmission factors were found in pulp tissues and periapical lesions (periapical abscesses, preapical granulomas, radicular cysts) [19,20]. It can be hypothesized that the virus is able to populate pulp tissues when access to it is possible due to pathological processes (e.g., caries) or a blood-borne pulp infection. Therefore, the infectivity for dental practitioners may be expected during dental procedures on healthy and disease-affected teeth in patients with SARS-CoV-2 infection. Likewise, periodontal interventions (see above) can produce SARS-CoV-2 transmission risks for dental professionals. Special preventive procedures should be implemented to prevent the spread of disease during oral cavity-related procedures.

## 4. Pathophysiology of the Oral Cavity in COVID-19

COVID-19’s clinical symptoms and signs can directly depend on infection of the oral cavity structural by SARS-CoV-2. It has been reported that the oral cavity tissues can be the first to be infected with SARS-CoV-2, and oral lesions could be the first manifestations of the disease. Therefore, dental practitioners could play an important role in the initial disease diagnosis, verified further by patient testing [43]. Invasion of oral mucosal cells, principally via the ACE2/TMPRSS2 mechanism, can affect the function of oral epithelial cells, finally resulting in ulcerated gingival lesions. However, the exact mechanism of the lesions is not established, and direct viral infection effects and/or severe systemic mechanisms are postulated to be involved. SARS-CoV-2 infection and replication is associated with inflammation and local immune cell activation. The most common histological finding is a chronic sialadenitis, also involving focal lymphocytic sialadenitis [10]. Immunohistochemistry images have demonstrated the predominance of T lymphocytic inflammation (CD3) with proportionally more B lymphocytes (CD20) in cases with SARS-CoV-2 focal lymphocytic sialadenitis [44]. It can be hypothesized that the infection of serous acinar cells in the parotid and submandibular glands and oral mucosa entails direct cell damage, along with triggering of a local inflammatory reaction leading to salivary gland dysfunction and xerostomia as a clinical consequence.

Elevated levels of proinflammatory cytokines such as IL-1β and TNF-α detected in inflamed gingiva in COVID-19 can confirm the local inflammatory state [41]. The disturbed immune system function induced by SARS-CoV-2 infection can favor the expansion of periodontal pathogens in periodontal pockets, e.g., *Prevotella intermedia*, Streptococci and Fusobacterium, thus promoting the development of acute periodontal conditions [41,45]. A further sequence of events, similar to a vicious circle, can include the expansion of the SARS-CoV-2 entry and processing factors in human gingival fibroblasts induced by lipopolysaccharides derived from periodontal pathogens (e.g., *Porphyromonas gingivalis*) or inflammatory cytokines/mediators (IL1β, TNFα) (for details, see above) [23].

Another of COVID-19′s local signs and symptoms manifested in SARS-CoV-2-infected patients is a loss of taste, reported in about 40% of COVID-19-positive individuals [46]. In symptomatic individuals, the presence of SARS-CoV-2 RNA in the saliva was positively associated with patients’ self-reported “loss of taste”. A report of two patients with a high viral load in the saliva proved taste changes associated with significant epithelial (pCK+) cell infection in ACE2-expressing cells [10]. The expression of SARS-CoV-2 entry and transmission factors (ACE2 and TMPRSS2) was also shown in fungiform papillae taste cells [8], and direct evidence of SARS-CoV-2 infection of a subpopulation of specialized taste receptor cells, namely, PLCβ2-positive Type II cells (expressing ACE2), in the taste papillae was provided by Doyle et al. [47]. The replication of SARS-CoV-2 was revealed by in-situ hybridization in Type II cells (which contain the G protein-coupled taste receptors for bitter, sweet and umami taste stimuli), which strongly suggest that taste Type II cells provide a portal for viral entry that predicts vulnerabilities to SARS-CoV-2 in the oral cavity. The study also found a disruption of continuity and cell turnover of the fungiform papillae taste stem cell layer during infection and for up to 6 weeks after symptom onset. Therefore, the viral cytopathic effects produced within the papillae taste cells can lead to local destruction and a clinical manifestation of taste loss. Massive destruction of taste cells produces a long-lasting impairment of smell and taste, which was seen in 11% of patients in a 6-month follow-up survey without any recovery and in 30% of individuals with only partial recovery [48]. The normal recovery time of most patients is short, varying from 4 to 17 days.

Molecular functional differences between variants of SARS-CoV-2, e.g., Delta and Omicron, may explain the published information from various medical centers about fewer instances of taste loss in individuals infected by the Omicron variant. The differences in the SARS-CoV-2 variants’ entry pathways may at least partly explain the clinical findings on taste loss. The Omicron variant is less fusogenic when compared with the Delta variant, and uses a less efficient endocytosis entry pathway, where affinity to ACE2 plays a crucial role, whereas the Delta variant enters mainly via a more efficient mechanism, i.e., via host membrane-bound peptidases, mainly TMPRSS2 [49,50]. The poorer fusion activity of the Omicron variant can be also attributed to the difference in the S1/S2 junction furin cleavage site. As a consequence, the Omicron variant replicates more slowly than the Delta variant in cells overexpressing TMPRSS2 [50], with a possibly reduced cytopathic effect. However, this hypothesis requires further study.

## 5. The Oral Cavity and Diagnostic COVID-19 Aspects

Saliva from SARS-CoV-2-infected individuals, in both acellular and cellular salivary fractions, harbors SARS-CoV-2 virus particles, and their detection enables due consideration of saliva as a valuable diagnostic specimen. In comparison with nasopharyngeal swabs (a diagnostic standard for SARS-CoV-2) RT-PCR SARS-CoV-2 detection in saliva demonstrated 96.1% concordance with nasopharyngeal swabs, with a detection sensitivity of 95.7% and a specificity of 97.62% [37]. This study used self-collected saliva samples, which might be an advantage (over nasopharyngeal swabs) of the method due to its easy application when health system resources are limited. These observations were confirmed by other studies, revealing the close agreement of saliva in a head-to-head comparison with nasopharyngeal swabs [51]. Huber et al. [51] showed saliva sampling’s applicability and even superiority over nasopharyngeal swabs in children. Notably, in children, SARS-CoV-2 infections were more often detected in the saliva than in nasopharyngeal swabs (positive predictive value = 84.8%).

The RT-PCR/MALDI-TOF mass spectrometry-based assay to detect targeted amplicons is an alternative method to the standard RT-PCR SARS-CoV-2 detection in saliva specimens (and also in upper respiratory specimens). The detection platform demonstrated high diagnostic sensitivity and specificity, and has the potential to increase diagnostic capacity as well as complement standard nucleic acid amplification testing technologies [52].

In summary, saliva is a generally reliable specimen for the detection of SARS-CoV-2, since it is a readily applicable, non-invasive, easy method of collecting test specimens. SARS-CoV-2 saliva tests have been developed and approved by the Food and Drug Administration (FDA) with an Emergency Use Authorization. The reliability of saliva samples for SARS-CoV-2 detection can be improved by the application of standardized saliva collection devices, which would increase the sensitivity for detecting symptomatic and pre-symptomatic infections [53].

In the diagnostic process, a measurement of IgA antibodies against SARS-CoV-2, which constitutes the early specific humoral response (detectable 2 days after the onset of symptoms), could be considered. These antibodies persist in saliva for 49–73 days after the occurrence of symptoms [34]. The saliva exhibits also sustained IgG antibodies against SARS-CoV-2 in asymptomatic patients [10]. Systemic IgG antibodies may be maintained in COVID-19 patients for at least 12 months after symptom onset [54,55]. Less information is available about the long-term kinetics of mucosal antibodies, which would be relevant to investigate in follow-up studies. These antibodies can be used as potential diagnostic method targets for detection of virus exposure in the active and recovery disease phases. The conversion dynamics of antibody levels (IgM, IgA, IgG) to SARS-CoV-2 spike and nucleocapsid antigens in the saliva was also proposed as a useful tool for screening populations to detect low viral load exposures [56].

Moreover, salivary amylase may be a marker of SARS-CoV-2 infection of the salivary glands. It is released into the peripheral blood after cytolysis of serous acinar cells produced by the virus. Thus, there is a possibility that the peripheral blood amylase levels of COVID-19-infected patients could be used to determine the range of damage in salivary glands [57].

## 6. The Oral Cavity’s COVID-19 Therapeutic Potential

The oral cavity can be a site of early SARS-CoV-2 infection and therefore could play an important role in transmitting the virus to the lungs or the gastrointestinal tract via the saliva, as has been suggested for other microbial-associated diseases, such as pneumonia and inflammatory bowel disease. Infection of the oral structure by the virus triggers local inflammation, which may promote periodontal pathology. Aspiration of oral secretions associated with periodontal disease (containing microorganisms such as *P. gingivalis*, *F. nucleatum* or *P. intermedia*) can produce contamination and infection of the lower airways [45]. Likewise, cytokines (e.g., IL-1β and TNF-α) from diseased periodontal tissues can infiltrate the saliva and be aspirated, and thus cause inflammation or infection within the lungs [58]. Therefore, adequate oral hygiene can reduce the occurrence of inter-bacterial exchanges between the lungs and the mouth, decreasing the risk of respiratory infections and potentially post-viral bacterial complications [59]. Reduction of oral inflammation can also limit the SARS-CoV-2 infection-promoting microenvironment in periodontal pockets, and reduce cellular entry of the virus (see above). Poor oral hygiene also results in the formation of niches, which retain the virus (see above), being sites of favorable virus retention. Tight control through oral hygiene procedures can reduce viral load in the oral cavity. In fact, single clinical cases suggest improved oral care affected (decrease) the time of oral viral shedding [60].

There is also some preliminary clinical evidence that the application of oral antiseptics can contribute to elimination of SARS-CoV-2 from the oral cavity. The three most recommended oral antiseptics against SARS-CoV-2 with promising observations are povidone-iodine, hydrogen peroxide, and cetylpyridinium chloride. These agents were shown to decrease viral load in the saliva for up to 2–3 h post mouthwash in up to 50% of COVID-19 patients. Based on the evidence collected so far, it may be advisable to use antiseptics, especially prior to an oral examination/treatment, i.e., povidone-iodine 0.2%, cetylpyridinium chloride 0.05–0.07 or hydrogen peroxide 1% [61]. Similar to antiseptics, the general ingredients of toothpastes and mouthwashes may affect the spike protein–ACE2 interaction and TMPRSS2 protease activity [62]. In vitro assays detected the inhibitory effects of sodium tetradecene sulfonate, sodium N-lauroyl-N-methyltaurate, sodium N-lauroylsarcosinate, sodium dodecyl sulfate and copper gluconate on the interaction between receptor-binding domain of spike proteins and ACE2. Molecular docking simulations revealed that these agents could bind to the inhibitor-binding site of ACE2. This study also revealed the inhibitory effects of the abovementioned agents and tranexamic acid against the serine protease activity of TMPRSS2 [62]. In conclusion, oral hygiene with commonly available tools, e.g., toothpastes and mouthwashes, could prevent SARS-CoV-2 infection or play a role in the development of COVID-19 complication and the course of the disease.

Lastly, the possibility of using the oral mucosa as a target for oral vaccines against SARS-CoV-2 was explored, since the virus not only gains access to the host by mucosal routes but mucosal sites also seem to harbor SARS-CoV-2. The Sabin-1 poliovirus cDNA-based RPS (recombinant poliovirus Sabin 1) vector (RPS-CTP) system was evaluated as a vector system for the development of a safe and effective oral mucosal COVID-19 vaccine platform [63]. However, this RPS-CTP platform-based mucosal vaccine is in the early phase of development.

## 7. Conclusions

The available information demonstrates that the oral cavity structures (keratinized and non-keratinized mucosa and salivary gland epithelia) possess SARS-CoV-2 entry and transmission factors, including ACE2 and TMPRSS2, which are considered as the principal ones. Infection of cells endowed with the virus entry structures was confirmed by clinical observations and cell culture studies. However, the dynamics of the local infection spread in asymptomatic, pre-symptomatic and symptomatic individuals remains to be established. The SARS-CoV-2 infection of different cell subsets in the oral cavity can explain some clinical features of the disease, but additional studies are mandatory to establish the pathophysiological role of the virus. Saliva, both the cellular and acellular fractions, comprises detectable amounts of SARS-CoV-2 particles, which, when present in droplets, can transmit the infection to other individuals and potentially spread the virus to the respiratory and gastrointestinal tract. The clinical significance of the latter mechanism in the course of COVID-19 remains to be entirely elucidated. Further efforts should be directed to verify and establish a local pharmacotherapeutic approach targeting SARS-CoV-2 in the oral cavity. Preliminary clinical observations suggest that reduction of the viral load in the oral cavity can exert favorable effects on COVID-19’s clinical course.

## Supplementary Materials

The following supporting information can be downloaded at: https://www.mdpi.com/article/10.3390/ijms23031431/s1.
