# Supplementary material for: Oral Pathology in COVID-19 and SARS-CoV-2 Infection—Molecular Aspects
Source: Int J Mol Sci. 2022 Jan 27;23(3):1431. doi: 10.3390/ijms23031431 (PMC8836070; doi:10.3390/ijms23031431)
Supplement: Supplementary file 1 [file ijms-23-01431-s001.zip › ijms-1557606-supplementary.pdf]

The following protocol was applied for the search and selection of the references cited in the review

I. Methods

1. Search strategy

PubMed and Scopus databases were used to search the adequate keywords to build up the picture of molecular mechanisms involved in the oral pathology during SARS-CoV-2 infection, such as: SARS-CoV-2, COVID-19, oral virus infection, saliva, crevicular fluid, salivary gland, tongue, oral mucosa, periodontium, gingiva, dental pulp, ACE2, TMPRSS2, Furin, diagnosis, topical treatment, vaccine and the related words for references published until December 28th, 2021. Titles and abstracts were separately reviewed by two authors (A.D and M.D) to select potentially related articles. The full texts of articles that appeared confusing were evaluated to determine their appropriateness for inclusion.

2. Included studies

The studies reviewed in this article included information about molecular processes of SARS-Co-2 infection of the oral cavity structures.

3. Excluded studies

The articles with only abstract were excluded from this review. Studies which only presented clinical symptoms and signs aspects of COVID-19 were also excluded from the analysis.

4. Data extraction

Data from articles were extracted independently by two reviewers/authors.

5. Quality assessment

Quality of each publication was evaluated by two independent reviewers (A.D and M.D). This review addressed 5 domains: oral cavity as SARS-CoV-2 entry gateway, saliva as a potential transmission factor, pathophysiology of oral cavity in COVID-19, oral cavity and diagnostic COVID-19 aspects, oral cavity COVID-19 therapeutic potential.

6. Statistical analysis:

It was not possible to conduct a meta-analysis because there was not enough proper research studies on this subject.

II. Results

7. Study selection

Finally 63 articles were retrieved for the final analysis, from initial database searches. 8 review articles due to redundant information were excluded.

8. Study characteristics (Eligibility criteria)

According to inclusion criteria stated in the protocol, articles in English language without any date restriction were included. The search process in PubMed and Scopus was updated up to 28.12.2021. The title and abstract of the remainder of the search results were reviewed by both authors to exclude irrelevant studies. Full texts of the remainder of the citations were collected for further screening and data extraction. Included studies were mostly original articles, and published in 2020 and 2021 with the aim to describe molecular processes of SARS-CoV-2 infection in the oral cavity.
